# Ethanol sensitizes hepatocytes for TGF-β-triggered apoptosis

**DOI:** 10.1038/s41419-017-0071-y

**Published:** 2018-01-19

**Authors:** Haristi Gaitantzi, Christoph Meyer, Pia Rakoczy, Maria Thomas, Kristin Wahl, Franziska Wandrer, Heike Bantel, Hamed Alborzinia, Stefan Wölfl, Sabrina Ehnert, Andreas Nüssler, Ina Bergheim, Loredana Ciuclan, Matthias Ebert, Katja Breitkopf-Heinlein, Steven Dooley

**Affiliations:** 1 Department of Medicine II, Section Molecular Hepatology, Medical Faculty Mannheim, Heidelberg University, Theodor-Kutzer-Ufer 1–3, 68167 Mannheim, Germany; 2 Dr. Margarete Fischer-Bosch Institute of Clinical Pharmacology, Auerbachstr. 112, 70376 Stuttgart, Germany; 3 Department of Gastroenterology, Hepatology, and Endocrinology, Hannover Medical School, Hannover, Germany; 4 Institute of Pharmacy and Molecular Biotechnology, Heidelberg University, Heidelberg, Germany; 5 Eberhard-Karls University Tübingen, BG Trauma Center, SWI, Schnarrenbergstraße 95, 72076 Tübingen, Germany; 6 University of Vienna, Department of Nutritional Sciences, Molecular Nutritional Science, Althanstr. 14, UZA II, A-1090 Wien, Austria; 7 Roche Products Limited, 6 Falcon Way, Shire Park, Welwyn Garden City, AL7 1TW UK; 8 Present Address: Miltenyi Biotec GmbH, Friedrich-Ebert-Straße 68, 51429 Bergisch Gladbach, Germany

## Abstract

Alcohol abuse is a global health problem causing a substantial fraction of chronic liver diseases. Abundant TGF-β—a potent pro-fibrogenic cytokine—leads to disease progression. Our aim was to elucidate the crosstalk of TGF-β and alcohol on hepatocytes. Primary murine hepatocytes were challenged with ethanol and TGF-β and cell fate was determined. Fluidigm RNA analyses revealed transcriptional effects that regulate survival and apoptosis. Mechanistic insights were derived from enzyme/pathway inhibition experiments and modulation of oxidative stress levels. To substantiate findings, animal model specimens and human liver tissue cultures were investigated. Results: On its own, ethanol had no effect on hepatocyte apoptosis, whereas TGF-β increased cell death. Combined treatment led to massive hepatocyte apoptosis, which could also be recapitulated in human HCC liver tissue treated ex vivo. Alcohol boosted the TGF-β pro-apoptotic gene signature. The underlying mechanism of pathway crosstalk involves SMAD and non-SMAD/AKT signaling. Blunting CYP2E1 and ADH activities did not prevent this effect, implying that it was not a consequence of alcohol metabolism. In line with this, the ethanol metabolite acetaldehyde did not mimic the effect and glutathione supplementation did not prevent the super-induction of cell death. In contrast, blocking GSK-3β activity, a downstream mediator of AKT signaling, rescued the strong apoptotic response triggered by ethanol and TGF-β. This study provides novel information on the crosstalk between ethanol and TGF-β. We give evidence that ethanol directly leads to a boost of TGF-β’s pro-apoptotic function in hepatocytes, which may have implications for patients with chronic alcoholic liver disease.

## Introduction

Alcohol abuse is a major burden of Western countries. Excessive alcohol consumption may lead to fatty liver, fibrosis, and hepatitis. In many patients, continuous drinking causes cirrhosis, which significantly increases the risk for development of liver decompensation, hepatocellular carcinoma (HCC), and mortality^1,2^. Ethanol provokes an array of detrimental effects, systemically and on specific organs and cell types. Key features of alcoholic liver diseases (ALD) are upregulated oxidative stress levels, disturbed hepatocyte metabolism, increased influx of bacterial products to the liver (endotoxins, e.g., LPS), hepatic inflammation, and compromised regeneration as well as high mortality^2^.

Hepatocytes constitute the major cell type for the biotransformation of ethanol to acetaldehyde. For this, three major enzymatic systems are available^3^. In normal liver, alcohol dehydrogenase (ADH) is most relevant, using NADP^+^ as electron acceptor to oxidize ethanol to acetaldehyde. Additionally, catalase is capable of oxidizing ethanol. Finally, the microsomal ethanol oxidation systems (MEOS) accounts for rapid ethanol oxidation, with CYP2E1 being the key enzyme. Importantly, CYP2E1 is directly induced by ethanol. Ethanol metabolism increases reactive oxygen species (ROS) production, especially of H_2_O_2_ and superoxide anion (O_2_^−^), therewith weakening the cell’s defense system and causing direct cell damage, e.g., by lipid peroxidation^4–6^.

In the context of hepatocyte functionality, a key effect of ethanol is promotion of steatosis by increased fatty acid biosynthesis (via fatty acid synthase) and disturbed fatty acid oxidation (β-oxidation). Finally, it is well accepted that ethanol contributes to hepatic insulin resistance^7^.

Transforming growth factor beta1 (TGF-β) is a key pro-fibrotic cytokine in a broad range of chronic liver diseases and is abundant in ALD^8–13^. TGF-β dimers bind to receptor complexes (TGF-β receptors II and I) at the cell surface to initiate intracellular signaling cascades. The canonical pathway starts with C-terminal phosphorylation of SMAD2 and SMAD3 by TβRI, which enables interaction with SMAD4, forming a complex that translocates to the nucleus to regulate gene expression. Furthermore, several other pathways are triggered by TGF-β exposure in liver and other tissues, such as TAK1, PI3K/AKT, and small GTPase-dependent signaling networks^14,15^. TGF-β exerts a cell type specific response. It is a potent immune suppressor and mediates activation of quiescent hepatic stellate cells, therewith enhancing fibrogenesis. In epithelial cells, TGF-β may induce epithelial to mesenchymal transition (EMT), growth arrest, and promote apoptosis. In line with the latter, pro-apoptotic factors are induced by TGF-β in hepatocytes^16^, such as NOX4 leading to generation of ROS^17,18^ and other pro-apoptotic factors, such as BIM and BMF^19^. During liver regeneration, TGF-β accounts for ceasing of hepatocyte proliferation^16,20,21^.

Owing to the abundance of TGF-β in chronic liver diseases and the impact of ethanol on cellular integrity, we asked whether alcohol may modulate the hepatocyte’s response to this cytokine. In a previous investigation, we already showed that TGF-β signaling is induced in mouse livers after chronic ethanol insult as well as in human alcoholics. TGF-β treatment of mouse hepatocytes induced lipid-, oxidative stress metabolism-, and fibrogenesis-gene expression signatures. Interestingly, TGF-β downregulated expression of the alcohol metabolizing enzyme ADH1 via ALK5/SMAD2/3 signaling, thereby increasing alcohol-induced lipid accumulation and CYP2E1-dependent toxicity^22^. We now analyzed the molecular mechanism, by which TGF-β signaling and alcohol challenge integrate in cell fate decisions of hepatocytes. We found that TGF-β’s pro-apoptotic function is massively enhanced by ethanol. Noteworthy, this finding is not explained by increased SMAD signaling alone but rather by an alcohol-triggered communication with other signaling cascades leading to a distinct apoptosis-related gene expression signature. Curiously, this effect is not mediated by increased oxidative stress or ethanol’s oxidation to acetaldehyde, but rather involves an interference with AKT signaling and GSK-3β, which have previously been linked to apoptosis or survival, respectively^23^. In summary, we provide evidence that ethanol is a crucial modulator of TGF-β effects, which may have clinical implications in patients with pre-existing liver fibrosis even when consuming only moderate amounts of alcohol.

## Results

### Ethanol sensitizes culture-stressed hepatocytes for TGF-β-mediated apoptosis

**Fig. 1:**
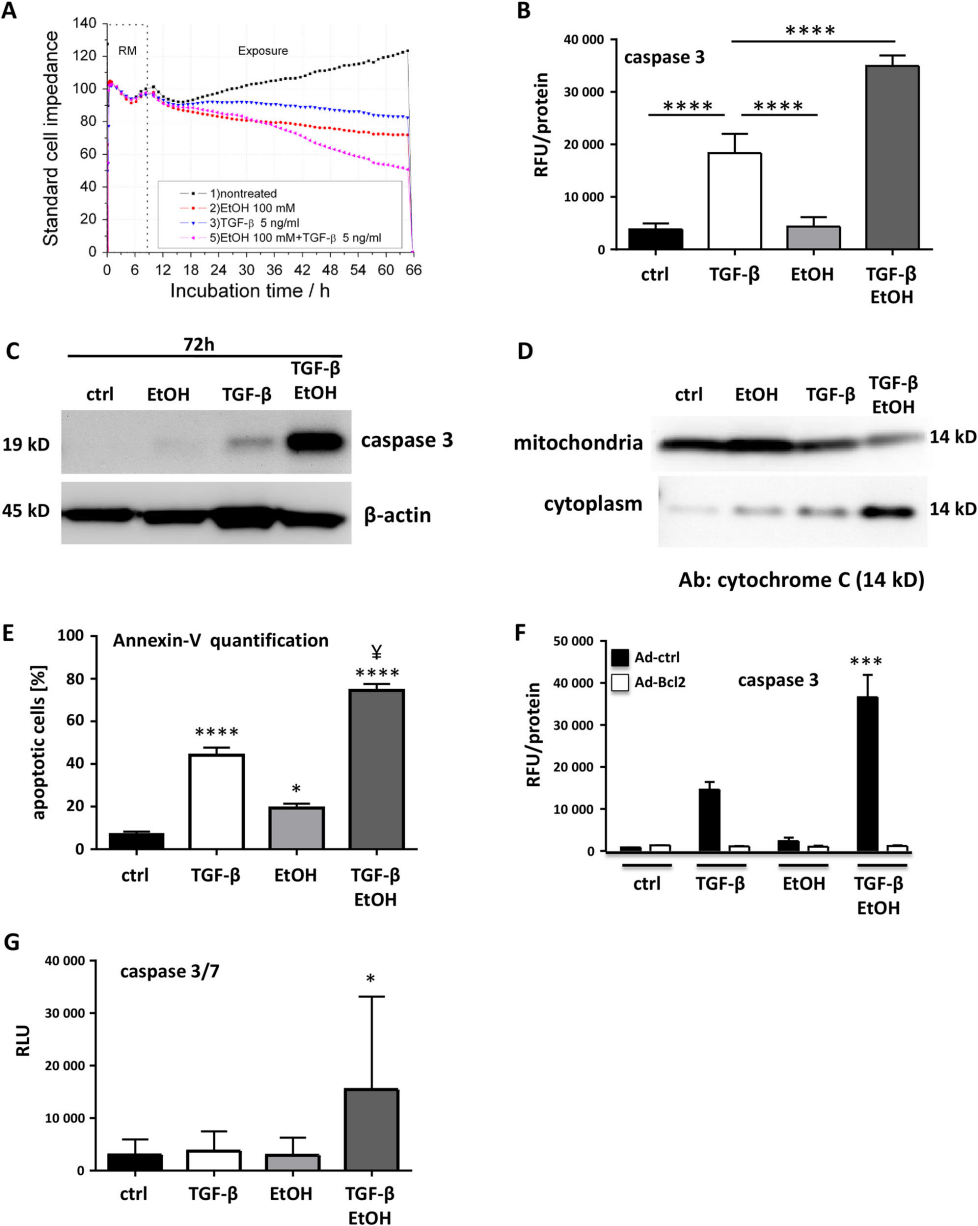
Ethanol plus TGF-β exert a strong apoptotic response in mouse hepatocytes and human HCC **a** Real-time response profile of cellular impedance upon ethanol/TGF-β treatment of mouse hepatocytes. Exposure to TGF-β (5 ng/ml) and/or ethanol (100 mM) started after 8 h equilibration of hepatocytes in the biosensor chip system and was continued over 58 h. Beginning of treatment is indicated by the dashed vertical line. RM = running medium (medium without substances). At beginning of treatment, RM is replaced by medium containing ethanol or TGF-β or both at the indicated concentrations. **b** Caspase-3 activity was measured in mouse hepatocytes stimulated with either TGF-β (5 ng/ml), ethanol (150 mM) or both for 48 h. The average activity of three independent experiments ± SEM is shown. Values were normalized to the total protein content of each sample. RFU = relative fluorescence units. **c** Detection of cleaved caspase-3 by immunoblot analysis using hepatocyte lysates after 72 h treatment as described for **b**. Detection of β-actin was performed as loading control. **d** Cellular lysates of mouse hepatocytes treated as in **c** were separated into cytoplasmic and mitochondrial fractions, and presence of cytochrome C was detected by immunoblot analyses. Ab antibody. **e** Mouse hepatocytes treated with 5 ng/ml TGF-β1, 100 mM ethanol or a combination of both were stained with Annexin-V-Cy3 and Hoechst 33342 (nuclei). Resulting fluorescent signals were analyzed using the ImageJ Software (particle analyzer). Relative amounts (in %) of apoptotic cells are shown (*N* = 9 pictures analyzed per group). *P*-values relate to control group. ^¥^ indicates *P* < 0.001 between TGF-β and TGF-β + EtOH group. Normal distribution was tested by Kolmogorov–Smirnov test, followed by one-way ANOVA with Bonferroni’s multiple comparisons test. **f** Hepatocytes were transfected with adenoviral vectors either expressing *LacZ* (Ad-ctrl) or *Bcl2* (Ad-Bcl2). 2 h after infection, viruses were removed, and after 2 h starvation, cells were stimulated with TGF-β and/or ethanol as indicated for 48 h. Caspase-3 activity was measured as in **b** and **c**. *** indicates a significant difference between LacZ + TGF-β and LacZ + TGF-β + EtOH, as measured by two-way ANOVA test, *P* < 0.001. **g** Effect of TGF-β, ethanol or both on caspase activation in extracts of human HCC tissues (*N* = 6). Combined stimulation with TGF-β and ethanol resulted in significantly increased caspase activation compared to all other treatment groups. Analyzed by one-way ANOVA, coupled with uncorrected Fisher’s least significant difference (LSD) test (in comparison to all other treatment groups; *P < 0.05)

As ethanol abundance affects cellular integrity and functionality, we aimed at investigating its consequences on TGF-β-mediated cellular phenotypes. Initially, we used a biosensor chip (BIONAS 2500, Bionas GmbH, Rostock) to follow real-time changes in cellular morphology, adhesion, cell–cell interactions, and membrane functionality of mouse hepatocytes via impedance measurement in response to ethanol and TGF-β (Fig. 1a)^24,25^. Although TGF-β and ethanol alone did only marginally reduce cell impedance, combined application of both effectively reduced cell impedance. Estimated values of the combined treatment started to deviate from single substance treatment at 30 h (22 h following exposure) and resulted in a loss of impedance at 66 h (58 h). Changes in impedance are a good indicator for the initiation of apoptosis^25,26^, but can also reflect other changes in cellular interaction and adhesion properties, including EMT^24^. Because TGF-β is known as initiator of epithelial cell apoptosis, we next measured caspase-3 activity (Fig. 1b). Interestingly, only TGF-β led to activation of caspase-3 in individual treatments after 48 h (Fig. 1b). This was further increased significantly after combined application of ethanol and TGF-β. This pro-apoptotic signaling was further confirmed using immunoblot (Supplementary Fig. 1a, c). The effect of ethanol was dose-dependent (Supplementary Fig. 1b). Investigating the apoptotic route responsible we found by immunoblotting that mitochondrial cytochrome C release was strongest with co-treatment (72 h, Fig. 1d), which further indicates that ethanol sensitizes hepatocytes for TGF-β-mediated apoptosis. Next, Annexin-V staining with Hoechst 33342 counterstain of nuclei was performed. In agreement with the former results (Fig. 1b), strongest positivity for Annexin-V was detected upon co-treatment (Fig. 1e; Supplementary Fig. 1c). BCL2 had been demonstrated to counteract TGF-β-mediated apoptosis^27^. Hence, we overexpressed BCL2 in mouse hepatocytes utilizing an adenoviral construct. BCL2 over-expression completely blunted TGF-β-triggered cell death and prevented ethanol-dependent super-induction (Fig. 1f; Supplementary Fig. 1d).

We next investigated the effect of TGF-β and ethanol in fresh liver tissue explants from human HCC (*N* = 6). No significant differences in caspase-3/-7 activity were found between HCC tissues treated with ethanol or TGF-β and untreated control. This is probably due to a more robustly transdifferentiated phenotype in HCC cells, where the anti-proliferative function of TGF-β is replaced by tumor promoting actions of the same pathway. However, as in cultured cells, co-treatment with TGF-β and ethanol lead to resensitization with significantly increased caspase-3/-7 activity (Fig. 1g). These results demonstrate that the apoptosis sensitizing role of ethanol on TGF-β signaling is relevant also in the tissue in situ. The importance of TGF-β signaling is underlined by elevated phospho-Smad levels already in untreated HCC tissues (Supplementary Fig. 1e, f). The possible therapeutic impact of this finding on therapy of HCC patients needs to be determined in another study.

### Ethanol- and TGF-β-mediated apoptosis gene expression signature in mouse hepatocytes

**Fig. 2:**
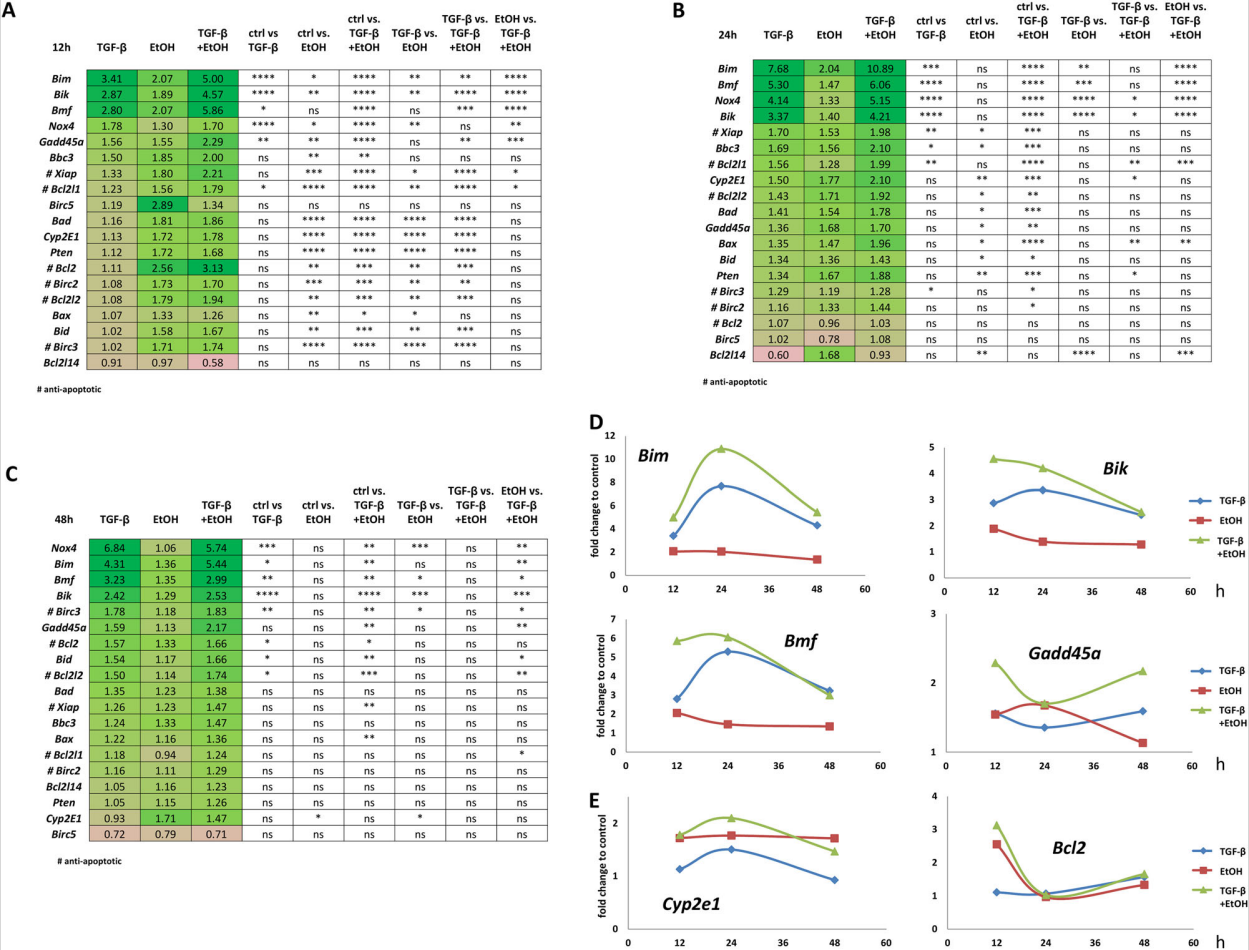
Ethanol/TGF-β-mediated pro- and anti-apoptotic gene signature **a**–**c** Mouse hepatocytes were treated with 5 ng/ml TGF-β, 150 mM ethanol, and both for 12 h (**a**), 24 h (**b**), and 48 h (**c**). Expression profiling was conducted with Fluidigm qPCR technology. Changes in expression are shown (fold) relative to untreated. The lists are sorted by TGF-β group descending in fold expression. Analyses were conducted with one-way ANOVA with multiple comparison and Tukey correction. ns. not significant; # anti-apoptotic genes. **d** Time course analyses (assembled from data of **a**–**c**) of strongly altered pro-apoptotic genes (*Bim, Bik, Bmf*, and *Gadd45a*). For all apoptosis-promoting targets, co-treatment of TGF-β and ethanol triggered strongest induction. **e** Time-resolved pattern of ethanol metabolizing enzyme *Cyp2e1* and the anti-apoptotic gene *Bcl2* (data derived from **a**–**c**)

To more comprehensively delineate pathway components participating in the TGF-β apoptotic response of mouse hepatocytes upon sensitization with ethanol, we assessed the transcription profile of a selected panel of pro- and anti-apoptotic genes 12, 24, and 48 h after treatment, using the Fluidigm real-time PCR platform. Strongest changes occurred at early time points (Fig. 2a, b). Compared to single treatments, a substantial fraction of target genes (including *Bim*, *Bik*, and *Bmf)* was significantly increased by co-treatment. Noteworthy, although several anti-apoptotic genes were also regulated, pro-apoptotic factors were stronger induced at all time points. At 48 h, ethanol alone did not affect apoptotic genes any more, except for increased *Cyp2e1* expression (Fig. 2c). In contrast, TGF-β still kept pro-apoptotic factors at higher expression levels, e.g. *Nox4*, *Bim*, and *Bmf*, which was more pronounced in the presence of ethanol. These changes over time and the boosting effect of ethanol on TGF-β target gene expression is illustrated in Fig. 2d for *Bim*, *Bik*, *Bmf*, and *Gadd45a*. At 12 h, super-induction from co-treatment is evident for all targets, whereas at 24 h, this effect only increased further for *Bim*. After 48 h, only *Gadd45a* was still super-induced, but did not reach significance any more (Fig. 2c). With regard to known ethanol target genes, *Cyp2e1* was increased at all time points and anti-apoptotic *Bcl2* only at 12 h (Fig. 2e). Upon co-treatment, TGF-β did not further increase expression of these ethanol target genes, indicating that the synergistic effect only occurs for TGF-β-regulated genes, but not vice versa. To summarize, ethanol is a potent sensitizer for the TGF-β-regulated apoptosis-related gene expression program in hepatocytes, leading to super-induction, especially at early time points (12 and 24 h).

### Ethanol sensitizes hepatocytes for TGF-β signaling by upregulating TβRII expression

**Fig. 3:**
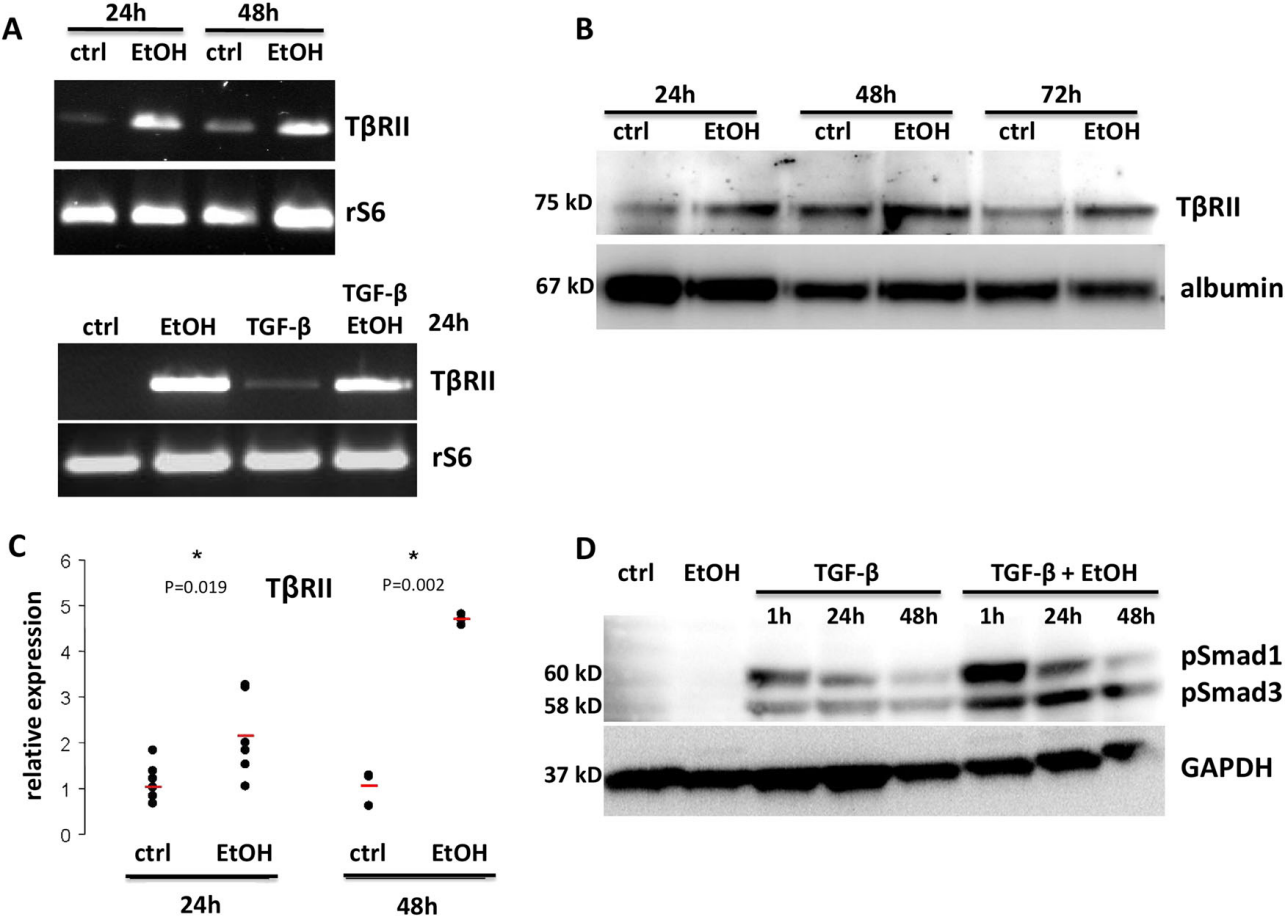
Ethanol induces TβRII expression in mouse liver and cultured hepatocytes **a**–**c** Mouse hepatocytes were incubated with ethanol (150 mM) for 12, 24, 48, and 72 h, as indicated. Subsequent mRNA analyses (conventional PCR, **a**) and immunoblot analyses (**b**) revealed significant induction of TBRII (most pronounced at the earliest time point; *P* < 0.05 for all time points, two-sided unpaired *t*-test). **c** Livers from mice having received an alcohol binge were isolated 12 and 48 h post gavage. mRNA expression of *TβRII* was significantly elevated at both time points (two-sided unpaired *t*-test). **d** Immunoblot analysis of TGF-β-treated (time as indicated, 5 ng/ml) or -untreated (ctrl) mouse hepatocytes, pretreated or not with ethanol (48 h, 150 mM), showing phospho-SMAD1 and phospho-SMAD3 levels and GAPDH as control

**Fig. 4:**
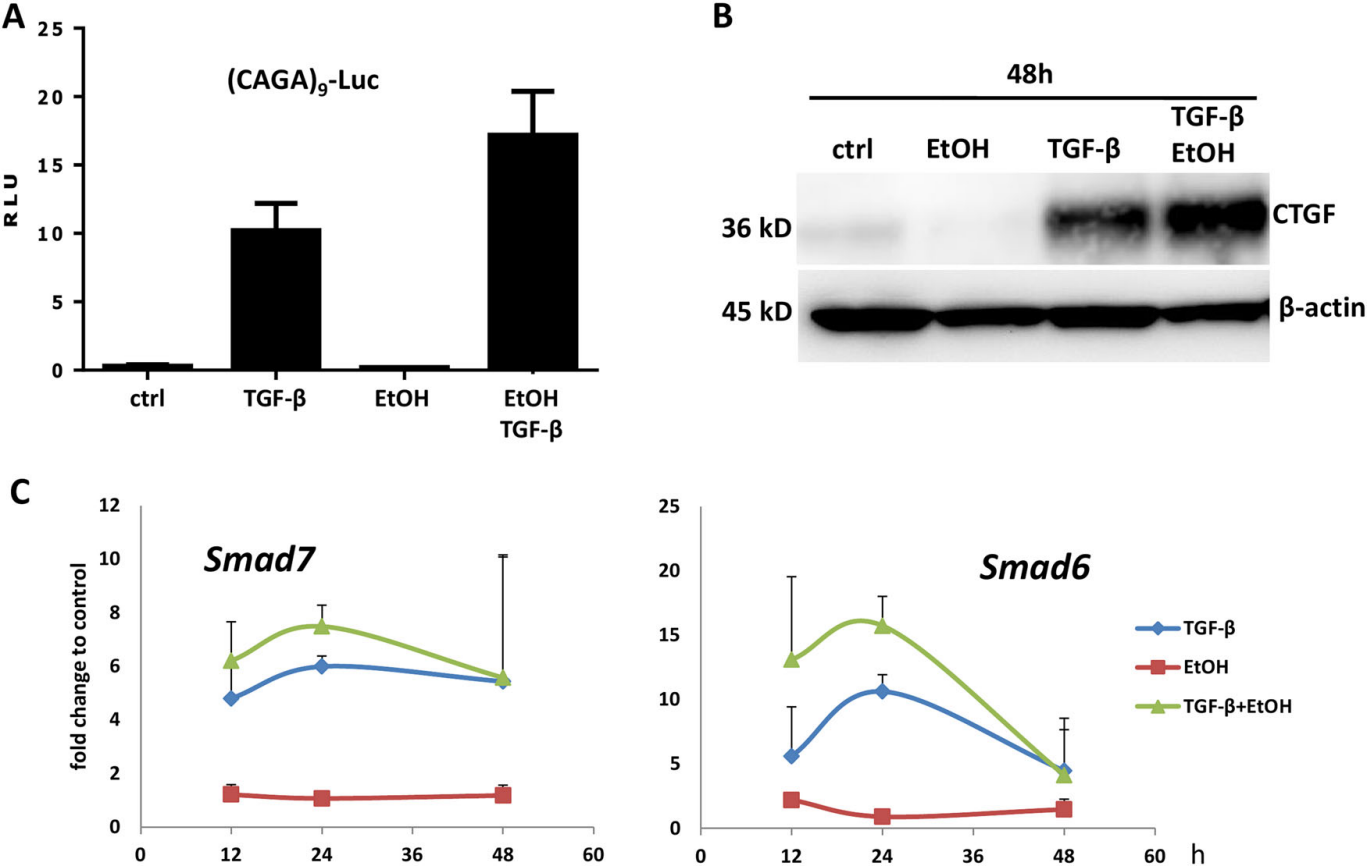
**a** TGF-β/SMAD3 signaling reporter assay using Ad(CAGA)9-MLP-Luc infection of mouse hepatocytes (for 2 h during attachment phase) and TGF-β/ethanol (5 ng/ml; 150 mM) treatment, as indicated. Obtained values (relative light units, RLU) were normalized to protein content. The experiment was performed in triplicates. **b** Immunoblot analysis of CTGF expression and **c** real-time RT-PCR analyses of *Smad6* and *Smad7* expression in mouse hepatocytes treated with TGF-β and ethanol as indicated and described in Fig. 1b

Next, we tested whether ethanol directly modulates the TGF-β signaling pathway in mouse hepatocytes. In time course experiments, we found mRNA and protein levels of TβRII significantly increased upon ethanol treatment (direct TGF-β-induced upregulation was very mild), as measured by conventional RT-PCR (Fig. 3a), real-time PCR (Supplementary Fig. 2a), and immunoblotting (Fig. 3b). In mice, ethanol intoxication similarly significantly induced TβRII expression in liver tissue, 12 and 24 h post gavage (Fig. 3c). These data indicate that acute alcohol intoxication induces expression of rate limiting components of the TGF-β signaling pathway, thereby enhancing responsiveness of the cells. This was further supported by significantly increased phosphoprotein levels of SMADs −1 and −3 in cultured mouse hepatocytes upon TGF-β treatment when the cells were pretreated with ethanol (Fig. 3d, Supplementary Fig. 2b). In line with this finding, TGF-β downstream signaling via SMAD3/4 was enhanced by ethanol co-treatment, as shown in reporter assays upon adenoviral infection of cultured mouse hepatocytes with a SMAD binding element (CAGA) luciferase reporter construct (Fig. 4a).

Finally, co-treatment significantly increased canonical TGF-β target gene expression, as measured at the protein level for CTGF (Fig. 4b, Supplementary Fig. 2c) and at the mRNA level for *Smad6* and *Smad7* (Fig. 4c). Thus, ethanol potently enhances the TGF-β signaling cascade in mouse hepatocytes.

### Ethanol affects survival-signaling networks in hepatocytes and redirects TGF-β signaling towards apoptosis

**Fig. 5:**
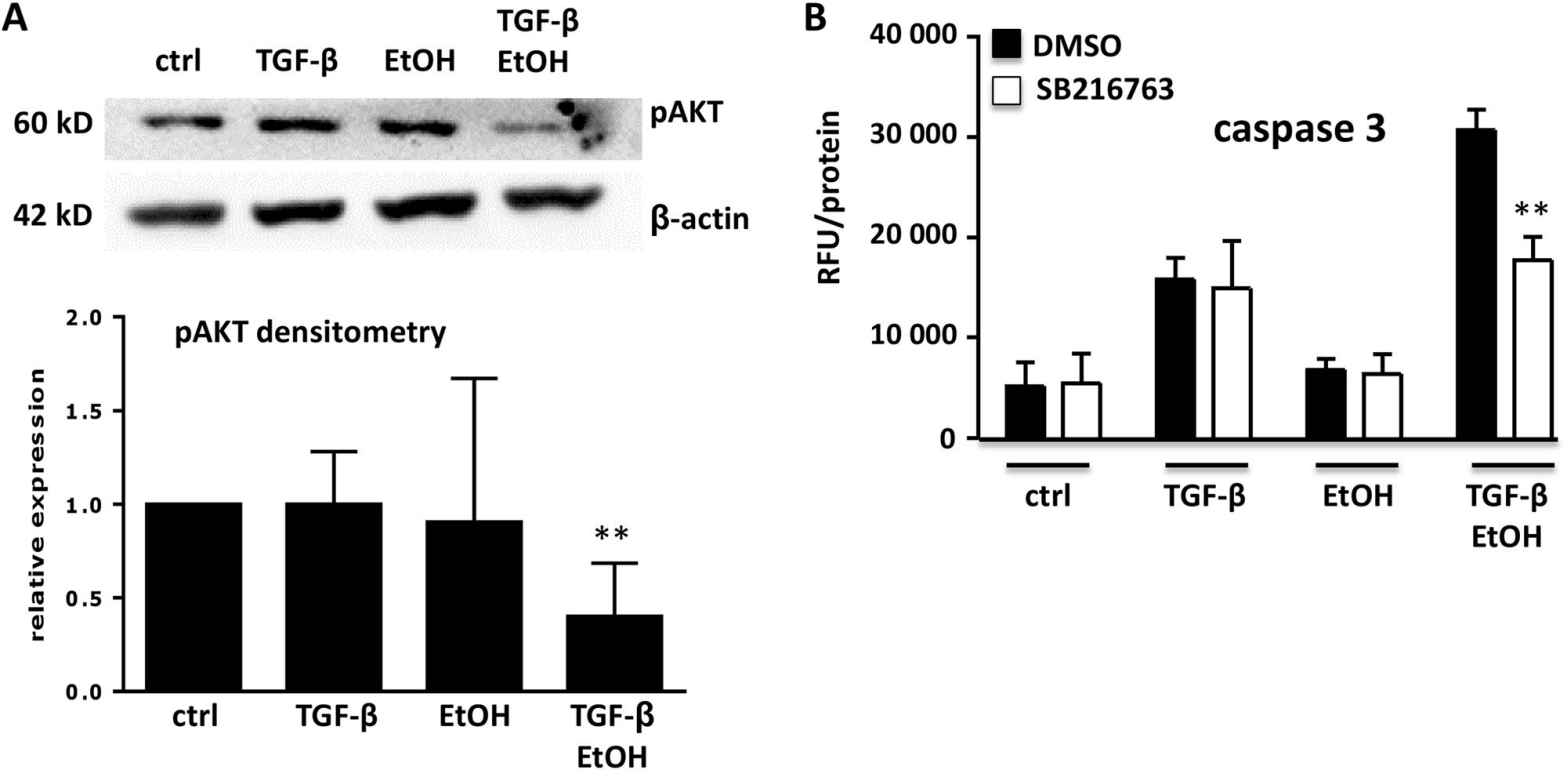
AKT/GSK-3β involvement in hepatocyte apoptosis **a** A representative immunoblot of TGF-β (5 ng/ml)- and ethanol (150 mM)-treated murine hepatocytes (48 h) shows alterations in phospho-AKT levels upon treatment, normalized (to GAPDH). Densitometric analyses of *N* = 6 experiments indicates a significant (*P* < 0.01) reduction in AKT phosphorylation (Ser473) in the TGF-β/EtOH compared to the untreated group (two-tailed *t*-test). **b** The GSK-3β inhibitor SB216763 (10 µM) was used to study the influence of GSK-3β on TGF-β- and ethanol-triggered apoptosis. A caspase-3 assay revealed that only upon co-treatment, blockage of GSK-3β reduced programmed cell death in murine hepatocytes (treatment for 48 h, *P* > 0.001, two-tailed t-test). Co, DMSO control; SB216763, 10 µM

Culture of mouse hepatocytes in 2D as monolayer represents a cellular stress situation with upregulated survival signaling and downregulated cytostatic responsiveness^28–30^. Moreover, thorough expression profiling indicated that this culture condition phenocopies cellular stress in mouse models and human patients with liver diseases^29^. Owing to our finding that in combination, ethanol challenge and TGF-β signaling are central in twisting the balance of the homeostasis of the hepatocyte phenotype towards a higher sensitivity for cell damage, we speculated that the survival pathway via AKT might be involved. We and others have previously shown that AKT activity is crucial for resistance to TGF-β-mediated apoptosis^31,32^. Co-treatment demonstrated significant abrogation of AKT phosphorylation at Ser473 (*P* < 0.01, *N* = 6)—a finding not observed by single treatments (Fig. 5a), with unchanged total AKT levels (Supplementary Fig. 3a). Using the PI3K/AKT small molecule inhibitor LY294002 in two different concentrations, we could phenocopy the ethanol effect on sensitizing mouse hepatocytes for apoptotic TGF-β signaling (Supplementary Fig. 3b). AKT is a negative regulator of GSK-3β and thus blocking GSK-3β function using a chemical inhibitor (SB216763) significantly reduced caspase-3 activity upon co-treatment (Fig. 5b, *P* = 0.005). Noteworthy, SB216763 neither affected basal nor direct TGF-β or ethanol induced apoptosis (also Supplementary Fig. 3c). Thus, AKT/GSK-3β signaling is pivotal in mediating the synergistic TGF-β and ethanol triggered hepatocyte death. In line with this, PTEN, a negative regulator of AKT activation was directly induced by ethanol treatment (Supplementary Fig. 3d).

### Ethanol, but not acetaldehyde, triggers the apoptotic effect

**Fig. 6:**
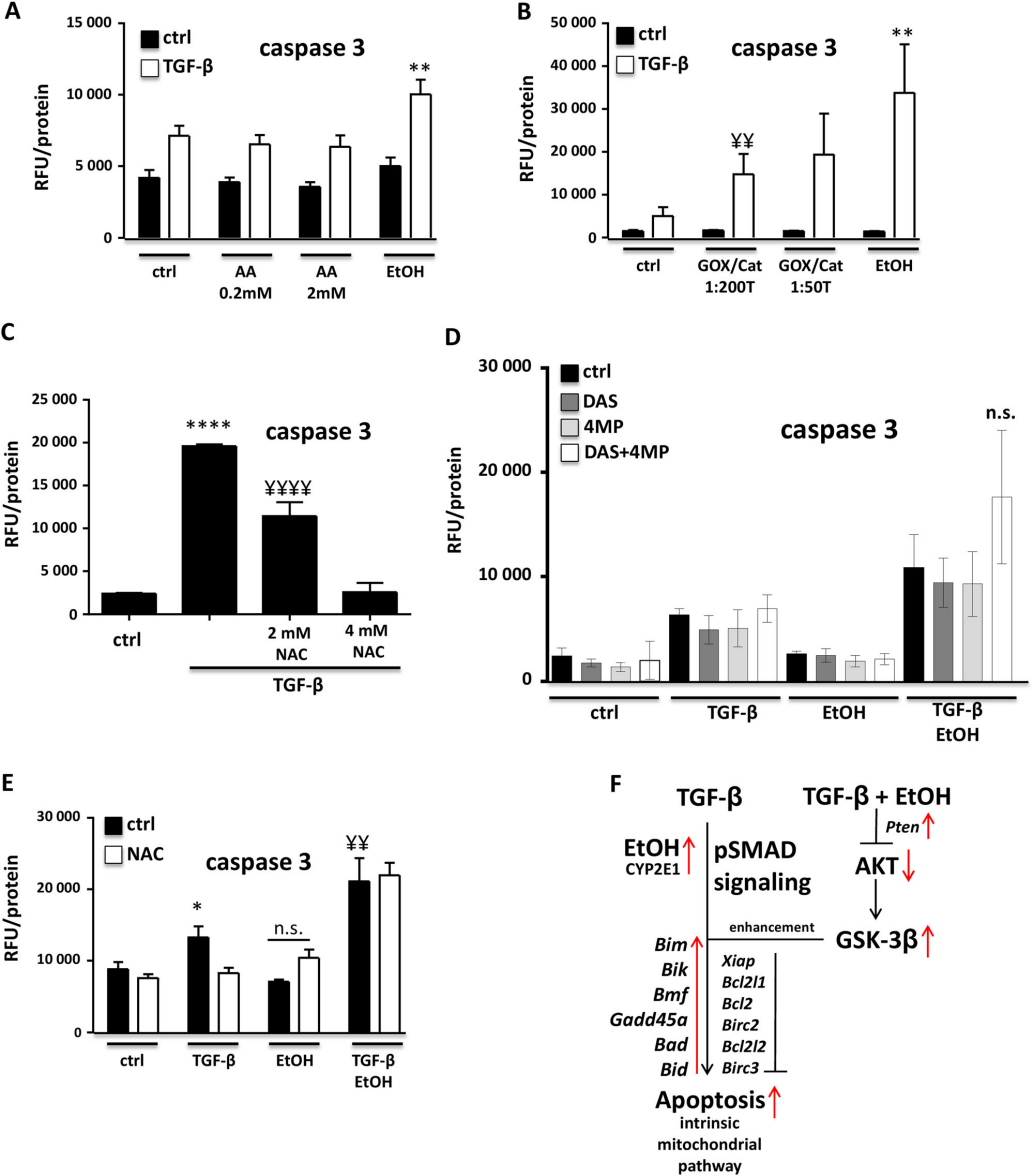
**a** The ethanol metabolite acetaldehyde does not affect TGF-β-mediated apoptosis in murine hepatocytes. Co-treatment of TGF-β (5 ng/ml) with ethanol (150 mM) led to enhanced hepatocyte apoptosis (*P* < 0.01). Its metabolite acetaldehyde was tested with 0.2 and 2 mM in combination with TGF-β for 48 h. Caspase-3 activity was not altered compared to only TGF-β treatment (ANOVA with multiple comparison and Tukey correction). **b**–**e** The role of oxidative stress in ethanol’s enhancement of apoptosis. **b** Caspase-3 assay was performed to analyze the effect of oxidative stress on TGF-β-mediated apoptosis. Increased activity of GOX (glucose oxidase) leads to production of reactive oxygen species in the cell medium, with catalase (CAT) supplementation balancing this effect. Reduced catalase levels led to increased oxidative stress, and upon TGF-β (5 ng/ml) addition, a GOX dose-dependent effect on caspase-3 activity was measured. ^¥¥^*P* < 0.01 indicates comparison of GOX/CAT 1:200,000 + TGF-β vs. no-GOX/no-CAT + TGF-β. TGF-β + GOX is highly significant compared with all other treatment groups (one-way ANOVA with multiple comparison and Tukey correction). **c** Supplementation of the antioxidant N-acetylcystein (NAC, 2 and 4 mM) significantly reduced TGF-β-mediated apoptosis, as measured by caspase-3 activity (48 h treatment). * indicates comparison with untreated group, ^¥^ indicates comparison with TGF-β only treatment. 4 mM NAC supplementation entirely blunted TGF-β-mediated apoptosis in murine hepatocytes (one-way ANOVA with multiple comparison and Tukey correction). **d** Blocking ADH (with 4-MP, 0.75 mM) and CYP2E1 (with DAS, 0.5 mM) enzymatic activities did not affect ethanol (150 mM)-mediated elevation of TGF-β-triggered hepatocyte apoptosis, as measured by caspase-3 activity (after 48 h). No significant difference was found in the TGF-β + ethanol group with inhibitor treatments. **e** NAC treatment (4 mM) in the context of TGF-β and ethanol-triggered apoptosis (48 h) revealed that the ethanol boost in programmed cell death is not blocked by reducing oxidative stress. ^¥^ indicates a highly significant difference between TGF-β treatment and TGF-β + ethanol treatment. NAC blunted TGF-β-mediated apoptosis, as also shown in **c** (one-way ANOVA with multiple comparison and Tukey correction). **f** Schematic illustration of ethanol’s mode of action on TGF-β-mediated apoptosis. Ethanol co-treatment leads to downregulation of the pro-survival AKT pathway, which enables GSK-3β functionality. This event then leads to enhancement of apoptosis. In parallel, ethanol affects TGF-β pathway components, which may also enhance apoptotic signaling. TGF-β/SMAD signaling regulates anti- and pro-apoptotic genes, although the latter are stronger induced and push the balance towards programmed cell death. Oxidative stress is not involved in ethanol’s modulation of TGF-β-triggered apoptosis

Owing to the rapid metabolism of ethanol to acetaldehyde and the known toxicity of this metabolite, we aimed at exploring whether the apoptotic phenotype is a direct effect of ethanol or is due to its conversion to acetaldehyde. Treating hepatocytes directly with 0.2 and 2 mM acetaldehyde instead of ethanol, did not increase TGF-β-mediated induction of caspase-3 activity (Fig. 6a). This indicates that the super-induction of hepatocyte death is directly mediated by ethanol itself. To further support this hypothesis, we investigated whether generation of oxidative stress might have a role in this phenomenon.

### Oxidative stress and ethanol metabolism

It is well accepted that oxidative stress is an essential component of TGF-β-mediated apoptosis. Furthermore, *Nox4*, a TGF-β target gene, has previously been documented as trigger for hepatocyte apoptosis^17,18^. Hence, we applied an enzymatic system, which generates a stable level of reactive oxygen species, the so called GOX/CAT system^33^. With that, we could demonstrate that indeed increased oxidative stress is able to amplify TGF-β-induced caspase-3 activity (Fig. 6b). Looking for gene expression effects, we found that GOX/CAT treatment induced *TβRII* and *Cyp2E1* expression and blunted TGF-β-induced expression of the survival gene *Bcl2* (Supplementary Fig. 4). Furthermore, interfering with oxidative stress formation by application of N-acetylcysteine (NAC) yielded in reduced caspase-3 activity upon TGF-β exposure (Fig. 6c). As ethanol metabolism increases ROS levels, we blocked the major ethanol metabolizing enzymes, namely ADH and CYP2E1 with the inhibitors 4-MP^22^ and diallyl sulfide (DAS), respectively. Interestingly, neither blocking ADH or CYP2E1, nor their combined inhibition reduced ethanol-mediated hyperactivation of caspase-3 upon TGF-β exposure (Fig. 6d). Finally, we assessed the consequences of NAC on apoptosis super-induction. As shown before (Fig. 6c), TGF-β-mediated apoptosis was abolished in the presence of NAC, but NAC did not affect apoptosis induction by co-treatment (Fig. 6e). In conclusion, ethanol triggers TGF-β-mediated apoptosis neither by formation of its metabolite acetaldehyde nor via the production of oxidative stress, suggesting that the observed switch of TGF-β signaling from survival to death is a direct effect of alcohol, whereby mechanistic details still largely remain undetermined.

## Discussion

We describe a detrimental crosstalk between TGF-β signaling and ethanol exposure. Such scenario is relevant for alcohol patients with existing fibrosis, being frequently associated with increased TGF-β levels. Previously, we showed that cell damage-mediated lactate dehydrogenase release increases by such co-treatment due to elevated oxidative stress^6,34^. Here, we found that mouse hepatocytes underwent apoptosis upon co-treatment, whereas ethanol alone had no effect. Super-induction of apoptosis was mediated by mitochondrial cytochrome C release, supporting our previous results^22^, and confirmed by the blunting effect of BCL2^35^ on TGF-β and co-stimulation triggered apoptosis. We, however, do not take into account crosstalk with other resident liver cells or hepatocyte organization in lobular structures. Monolayer hepatocytes present with cellular stress and rapid dedifferentiation, reflecting a damage and plasticity fate, as present in human and mouse liver diseases, including liver cancer^36^. We further observed that stressed hepatocytes display activated survival signaling pathways, including ERK and AKT^28,30^. This is a prerequisite for switching TGF-β signaling from cytostatic to tumorigenic in the liver. The pro-apoptotic-signaling branch is abrogated and the alternate tumorigenic pathway is activated, comprising production of secreted growth factors for tumorigenic stroma formation, e.g., CTGF, PDGF, and VEGF^37–40^ and enhanced growth factor receptor expression supporting cell survival. In addition, gene expression changes occur for epithelial to mesenchymal transition, including upregulated snail and vimentin, and decreased ZO-1 and E-cadherin^41–43^, a TGF-β response previously summarized as late TGF-β signature and that significantly correlated with bad prognosis in HCC patients^44^.

To translate our in vitro findings to human disease, we analyzed liver tissue explants from HCC patients for the apoptotic response to TGF-β and ethanol. Noteworthy, in HCC tissue co-treatment induced the strongest response, indicating that this mechanism is conserved in primary tissue as well. Although being aware that TGF-β effects in HCC in principle are pro-tumorigenic, the effect of ethanol on cell death was obvious and may hint for a mechanism switching back the TGF-β pathway to cytostasis. Moreover, a therapeutic option for HCCs which cannot be treated by resection or radiofrequency ablation is the injection of pure ethanol. According to current knowledge, the mechanism of ethanol action is via triggering necrosis, partially by damaging small vessels with subsequent ischemia^45,46^. In our approach, we applied lower doses of ethanol, and found the in vitro described synergism with TGF-β is valid in HCC tissue as well, inducing cancer cell apoptosis. In high dose treatments, this may have implications at tumor margins, where only low concentrations of ethanol are reached.

To gain more insight on transcriptional regulation by ethanol, we screened for gene expression changes of the TGF-β pathway components. *TβRII* was upregulated by ethanol in vivo and in vitro. These observations point to a direct stimulating function of ethanol on the TGF-β pathway. Indeed, it has been shown that acetaldehyde, the major metabolite of ethanol, can trigger *TβRII* expression in hepatic stellate cells and can also cause release and activation of latent TGF-β1^47–49^. A positive regulation of the TGF-β signaling cascade by ethanol is also concluded from our analyses of SMAD2/3 phosphorylation, SMAD binding element reporter activation, and CTGF expression. CTGF is known to foster fibrotic processes and is a direct target of TGF-β^50,51^. Although we found highest induction of CTGF upon co-treatment with TGF-β and ethanol, we do not have evidence that other pro-fibrotic events in hepatocytes were regulated similarly (e.g., enhanced expressions of Snail, Col1a1, or Vimentin; data not shown). Elevated expression was also determined for *Smad6* and *Smad7*. These inhibitory SMADs provide negative feedback on the TGF-β/BMP-pathways^52^. Noteworthy, in addition to enhanced Smad2/3 phosphorylation, we observed elevated activation of Smad1 by TGF-β upon ethanol pretreatment. Although Smad1 is the classical mediator of the canonical BMP signaling pathway, it can also be activated by TGF-β, especially at very early time points^53^. Besides TGF-β signaling components, we addressed whether the ethanol induced pro-apoptotic phenotype is accompanied by changes in expression of pro- and anti-apoptotic genes. Indeed, especially after 12 and 24 h, a super-induction of apoptosis-related genes was found in the co-treatment cohort. Although at 48 h, this effect had mainly faded out, the apoptotic phenotype was still manifest. As this analysis handled gene expression data, a conclusion on protein expression or activation status could not be drawn. Yet, the alcohol effect is evident at early time points, therewith paving the way for abrogated cell survival and increased TGF-β-mediated apoptotic rates of the hepatocyte population. Execution of apoptosis is balanced by pro- and anti-apoptotic factors^35^. Thus, it is not surprising that also anti-apoptotic genes are partially upregulated by TGF-β and alcohol in our experimental setting, but in summary, the equilibrium got dysbalanced in favor of apoptosis execution.

As non-SMAD pathways have fundamental roles in mediating TGF-β effects, especially on cell survival, we analyzed AKT and ERK signaling pathway activities. ERK activity was not affected by ethanol treatment (not shown), but a significant reduction in AKT Ser473 phosphorylation was identified in co-treated hepatocytes. A major role of AKT interference on directing TGF-β signaling towards cell death has been acknowledged^31^, making it a likely candidate for mediating the super-induction of caspase-3. An interesting regulatory mechanism was demonstrated with AKT reducing NOX4 activity, thus counteracting programmed cell death^17^. Reduced AKT activity upon co-treatment might deplete such preventive mechanism. Downstream signaling events of AKT comprise inactivation of GSK-3β by Ser9 phosphorylation. GSK-3β has previously been linked to promoting apoptosis^23^ by controlling expression of a panel of apoptosis regulating factors. The targeted inhibition of GSK-3β activity by SB216763 yielded in loss of co-treatment triggered apoptosis super-induction. On the contrary, TGF-β-mediated apoptosis was not affected, providing strong support for the AKT/GSK-3β axis having a dominant role especially in ethanol’s enhancement effect on TGF-β-mediated cell death.

The question whether ethanol directly accounts for the rise in TGF-β-triggered death or whether this was rather executed by its metabolism was also addressed. As acetaldehyde was described as major cell-damaging effector upon ethanol oxidation, we replaced ethanol by acetaldehyde and tested the apoptotic response. Although, as discussed above, acetaldehyde is capable of triggering TβRII induction as well as TGF-β1 activation^48,49^, in our experimental setting, acetaldehyde did not cause increased hepatocyte death. Furthermore, next to excluding acetaldehyde as mediator, we could rule out a role of ethanol metabolism (and thus oxidative stress). Oxidative stress by its own increased TGF-β driven apoptosis and in a model with over-expression of CYP2E1, TGF-β exerted enhanced cell damage^54^. Further, stabilization of cellular stress defense capacity by addition of NAC protected against TGF-β driven programmed cell death. However, this treatment regimen could not overcome the co-stimulatory effect on apoptosis. The two major ethanol oxidizing systems, MEOS and ADH1, produce ROS. Thus, blocking the enzymatic systems by inhibitors should prevent alcohol-dependent production of reactive oxygen and acetaldehyde. Addition of these inhibitors did not prevent the co-treatment effect on hepatocyte survival, which further demonstrates that ethanol and not its metabolism is directly mediating the enhanced TGF-β phenotype.

In conclusion, we unravel a crosstalk of TGF-β signaling and ethanol in hepatocytes with detrimental consequences for survival (a schematic illustration is provided in Fig. 6f). We further show that this crosstalk integrates at the AKT/GSK-3β axis to interfere with cell dedifferentiation associated survival, thus sensitizing hepatocytes for TGF-β-induced cell death. We and others have previously shown that in liver, fine tuning of TGF-β downstream signaling via specific adaptor proteins and activity of survival signaling pathways triggers cellular fate and subsequent consequences for the organ. Mishra and coworkers have shown that the adapter protein β2 spectrin is required for SMAD3-mediated cytostatic TGF-β signaling in hepatocytes, and that its downregulation, e.g., by induced expression of the E3 ligase PRAJA and subsequent β2 spectrin ubiquitinylation and proteasomal degradation facilitates cancer stem cell development and HCC formation^55–57^. Similarly, in an investigation of enhanced liver damage upon combined HCV infection and alcohol consumption, loss of cytostatic TGF-β signaling was the critical step in cancer stem cell origination and HCC development. In detail, HCV infection induced TLR4 expression in hepatocytes, thus sensitizing hepatocytes for alcohol-induced LPS. Downstream signaling induced expression of the stemness factor Nanog, which in turn induces the oncogenes *Igfbp3* and *Yap1*. Both integrate at the TGF-β signaling pathway, YAP1 via stabilizing SMAD7 and IGFBP3 via activating AKT/mTOR signaling, together efficiently interfering with the cytostatic TGF-β response^58,59^. These findings underline that controlled cytostatic TGF-β signaling is required for hepatocyte physiology. On the other hand, recent work from Seki and colleagues indicates that counteracting survival signaling that dampens cytostasis is also necessary for cellular homeostasis in liver. In detail, they show that cell stress induced by high fat diet, similarly as in our setting with alcohol, results in enhanced TGF-β-mediated cell death. Furthermore, they found that high fat diet downregulates TAK1-mediated survival signaling, thus directing TGF-β signaling towards lipogenic gene expression and abrogation of the hepatocyte antioxidant response^60^. The group then fully depleted hepatocytes for TAK1 survival signaling in vivo and these mice rapidly developed aggressive liver cancer, which was completely rescued by additionally depleting hepatocytes of TβRII. In TAK1 knockout mice, TGF-β induced massive hepatocyte apoptosis, which in consequence resulted in strong inflammation and compensatory proliferation, together causing hepatocarcinogenesis^61^.

Thus, the presented data in the context of the current state of the art further indicate that the TGF-β signaling pathway has strong and critical impact on hepatocyte fate decisions that are directly related to liver diseases, including ASH, NASH, and HCC. Thereby, TGF-β downstream signaling is integrated into and controlled from a network of survival signaling pathways. Future studies need to shed light on the relevance of these findings in patients suffering from alcoholic liver disease and the possible consequences of continued drinking in a pre-damaged liver scenario with TGF-β abundance.

## Material and methods

### Reagents

Antibodies against pAKT (#4051), pGSK-3β (#9323), cytochrome C (#4280), and cleaved caspase-3 (#9664) were purchased from Cell Signaling (Leiden, Netherlands), pSMAD1/3 (#1880-1) was obtained from Epitomics (Abcam, Germany), GAPDH from SantaCruz (sc-47724, Heidelberg, Germany), and β-actin (#A5441) from Sigma (Taufkirchen, Germany). Secondary antibodies carrying HRP were obtained from SantaCruz. Diallyl sulfide (DAS), 4-Methylpyrazole hydrochloride (4-MP), N-Acetyl-l-cysteine (NAC), and SB216763 were from Sigma. Ethanol (absolute) and acetaldehyde were purchased from Merck (Darmstadt, Germany). TGF-β1 (“TGF-β”) was from Peprotech (Hamburg, Germany) and used at a concentration of 5 ng/ml. LY294002 was from Sigma-Aldrich and used as indicated.

### Cell isolation/culture/treatment procedures

Primary hepatocytes (from C57BL/6 mice) were isolated by collagenase perfusion technique as described elsewhere^28^. For cell culture experiments, cells were seeded as monolayer on collagen coated plates. Culture plates were pre-coated with a solution of 250 μg/ml rat tail collagen I, followed by UV light-mediated crosslinking^28^, yielding a thin layer of monomeric collagen. Williams E served as medium, supplemented during attachment phase (4 h) with Pen/Strep, l-glutamine (2 mM), dexamethasone (100 nM), and FCS (10%). After attachment, cells were treated with TGF-β or ethanol (50–150 mM as indicated) in Williams E medium supplemented with l-glutamine (2 mM) and Pen/Strep. Wells were covered with parafilm to reduce ethanol evaporation^62^.

### Adenovirus infection

The adenovirus encoding *Bcl2* was used at an MOI of 10 to prevent TGF-β-mediated cell death. As control, Ad*LacZ* was utilized at the same MOI. Cells were infected for 2 h during the attachment phase. Subsequently, viruses were removed, and cells were washed and used for stimulation experiments.

### Caspase-3 assay

A fluorimetric-based enzymatic caspase-3 assay was used as described previously^31^. In brief, cells were lysed and equal protein amounts were used for the assay. As caspase-3 substrate, AC-DEVD-AFC (Biomol, Hamburg, Germany) was used and after incubation for 1 h with cell lysate, fluorescence was measured (excitation 400 nm; emission 505 nm) with a Tecan infinite M200 (Thermo Scientific, Braunschweig, Germany) plate reader (96 well format). *Y*-axis in arbitrary units (relative fluorescence units; RFU, normalized to protein concentration).

### Annexin-V staining

As another technique to analyze hepatocyte apoptosis, Annexin-V staining was performed. After over night serum-starvation, mouse hepatocytes were treated with 5 ng/ml TGF-β1, 100 mM ethanol, or a combination of both for 24 h. Residual culture medium was washed off with PBS (2 mM CaCl_2_). Cells were stained for 5 min with 0.25 µg/ml Annexin-V-Cy3, 5 µg/ml Hoechst 33342, and 150 nM Sytox Green in PBS. Unbound stain was removed by washing with PBS (2 mM CaCl_2_) and fluorescent signals were detected immediately and analyzed using the ImageJ Software (particle analyzer).

### Cytochrome C release

To quantify cytoplasmic and mitochondrial cytochrome C, we used the Qproteome Mitochondria Isolation Kit from Qiagen (Hilden, Germany). Therewith, we were able to generate cytosolic and mitochondrial protein lysates. Subsequently, cytochrome C content was assessed by immunoblotting using a cytochrome C-specific antibody.

### SMAD luciferase reporter assay

The Ad(CAGA)9-MLP-Luc was used as a SMAD3 luciferase reporter (adenoviral infection for 2 h during attachment phase) to estimate SMAD transcriptional activity^63^. Luciferase substrate was purchased from Promega (Mannheim, Germany). Luminometric measurements were performed using Tecan infinite M200. Obtained values (relative light units, RLU) were normalized to protein content. Experiments were performed in triplicates. Shown are the means of three experiments.

### PCR (conventional and Fluidigm qRT-PCR)

Total RNA from hepatocytes was isolated using RNAeasy Mini Kit (Qiagen). Overall, 600 ng total RNA was reverse transcribed to cDNA with TaqMan Reverse Transcription Reagents (Applera GmbH, Darmstadt, Germany). Conventional PCR was performed as previously described^64^. For quantitative Real-time PCR, the Fluidigm Biomark high throughput qRT-PCR platform (Fluidigm Corporation, San Francisco, USA) was used with pre-designed taqman probes from Applied Biosystems, according to the manufacturer’s instructions^65^. Data were analyzed using the ΔΔCt method and expression values were normalized to the expression levels of *Gapdh* and β-actin. For each condition, 4–6 independent hepatocyte isolation sets were used (*N* = 6 for 48 h time point; *N* = 4 for 12 and 24 h time points). For each time point, four treatment groups were used (control, TGF-β, ethanol, TGF-β + ethanol). Expression changes upon treatment were calculated relative to the corresponding untreated control.

### Impedance measurement

Impedance was measured using a microfluidic flow biosensor chip system (BIONAS 2500, Bionas GmbH, Rostock, Germany) containing two interdigitated electrodes, which provide a measure of the cell layer permeability and changes in cell shape based on extracellular ion mobility (flux), using electrical properties of the cell layer as described earlier^24,25^. Real-time response profiling of cellular impedance upon ethanol/TGF-β treatment of primary mouse hepatocytes was performed as follows. Cells were seeded as monolayer at high density on the chip surface and were equilibrated in sensor chip culture medium with continuous medium exchange in 4 and 4 min (for equilibration) and 4 and 30 min (for long-term cultivation) flow/stop cycles. Exposure to ethanol/TGF-β started after 8 h of equilibration of hepatocytes in the biosensor chip system and was continued over 58 h. Beginning of treatment is indicated by the dashed vertical line. At beginning of treatment, RM (running medium without substances) is replaced by medium containing ethanol and TGF-β at indicated concentrations; control experiments are performed with RM; treatment is continued until the end of experiment after 66 h.

### Culture of liver explants and caspase-3/7 assay

We investigated freshly isolated, unfixed HCC tissues from HCC patients (*N* = 6) who underwent partial hepatectomy. This study was approved by the ethics committee of Hannover Medical School. Briefly, HCC explants were precisely divided into 125 mm^3^ cubes under sterile conditions and incubated with modified Eagle’s medium supplemented with TGF-β, ethanol, or both for 8 h. Following incubation, liver tissue was pulverized in liquid nitrogen and lysed with lysis buffer as described previously^66,67^. Tissue extracts were diluted in buffer containing 50 mM Tris-HCl (pH 7.4), 10 mM KCl, and 5% glycerol to reach a final protein concentration of 1 mg/ml. Activity of caspase-3/-7 was measured in duplicates by a luminescent substrate assay (Caspase-Glo; Promega, Mannheim, Germany). For this purpose, 10 µl of tissue extracts were incubated with 10 µl of caspase substrate DEVD-luciferin and luciferase reagent for 2 h at room temperature. Luminescence of the samples was measured in relative light units (RLU) by a luminometer (LB 960, Berthold, Bad Wildbad, Germany).

### GOX/CAT system

This system makes use of glucose oxidase (GOX) in the culture supernatant, producing reactive oxygen species (H_2_O_2_). To achieve a steady level, catalase (CAT) is added aiming at degrading reactive oxygen species. Enzymatic rates of the enzymes are described in ref.^33^. We used GOX with a 1:300,000 and CAT with a 1:50,000 or 1:200,000 dilution, as indicated.

### Acute alcohol intoxication—in vivo experiment

All procedures were approved by the local Institutional Animal Care and Use Committee (IACUC) and the Regierungspräsidium Stuttgart (application number V250/07 EM). Experiments were carried out in a facility accredited by the Association for Assessment and Accreditation of Laboratory Animal Care (AAALAC) at the University of Hohenheim. Female C57BL/6J mice (8 weeks old) received 6 g ethanol per kg body-weight (once) per oral gavage as previously described^68^. After 12 and 48 h, animals were sacrificed, the livers preserved and further processed for RNA isolation. Experiments were not blinded.

### Immunoblotting

Cell lysates were prepared using RIPA buffer (50 mM Tris-HCl (pH 7.5), 150 mM NaCl, 1% Nonidet P-40 (NP40), 0.5% sodium deoxycholate, 0.1% SDS, Proteases Inhibitor Cocktail, and Phosphatase Inhibitor Cocktail II (Roche, Mannheim, Germany))^69^. Protein concentration was determined using the DC protein assay (Bio-Rad). Up to 30 μg protein per sample were separated by SDS-PAGE (12%). Subsequently, proteins were transferred to a nitrocellulose membrane and blocked in 5% non-fat milk in TBST. Then, membranes were incubated with indicated antibodies (first antibodies overnight at 4 °C and second antibodies at room temperature for 1 h) and developed with ECL solution (Pierce) using Chemi-Smart imager (Vilber Lourmat, Eberhardzell, Germany). Aida Image Analyzer v. 4.25 was used to perform densitometric analyses.

### Data availability

The data sets generated during and/or analyzed during the current study are available from the corresponding author on reasonable request.

### Statistics

Analyses were performed using GraphPad Prism Software. Two-sided unpaired *t*-test was applied as indicated. Normal distribution of values was tested with the Kolmogorow–Smirnov test. Subsequently, one-way ANOVA tests were used with multiple comparison and Tukey’s correction or with uncorrected Fisher’s least significant difference (LSD) test. Shown are data of three experiments (or as indicated) with SEM. Significance: *P < 0.05, **P < 0.01, ***P < 0.001, and ****P < 0.0001.

## Electronic supplementary material

Supplemental Material
