# Supplementary material for: Ethanol sensitizes hepatocytes for TGF-β-triggered apoptosis
Source: Cell Death Dis. 2018 Jan 19;9(2):51. doi: 10.1038/s41419-017-0071-y (PMC5833779; doi:10.1038/s41419-017-0071-y)
Supplement: Supplementary file 1 — Supplemental Material [file 41419_2017_71_MOESM1_ESM.pdf]

## Supplemental Figures & Methods

### Figures

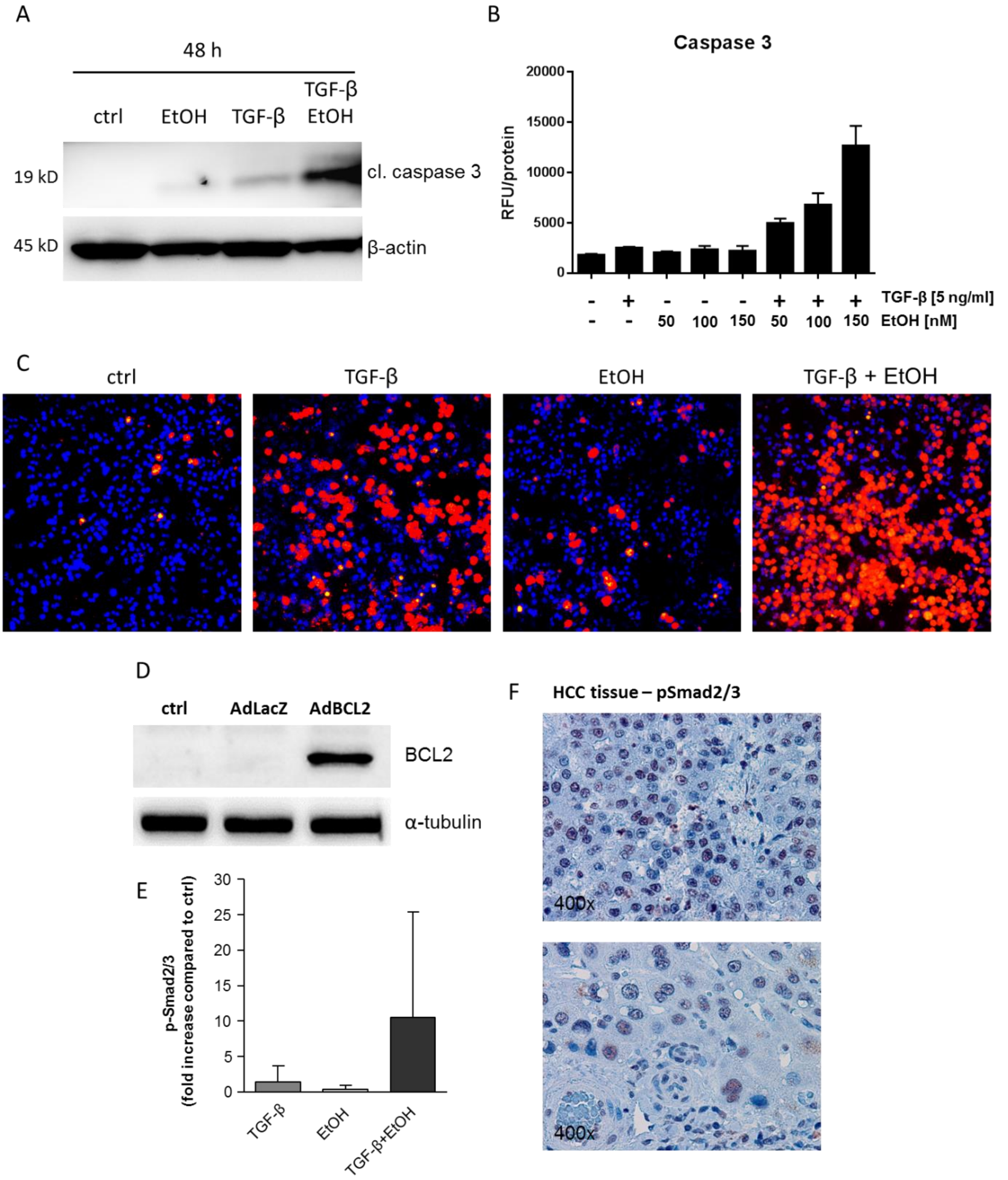


**Suppl. Figure 1**: (A) Detection of cleaved caspase-3 by immunoblot analysis using hepatocyte lysates after 48 h treatment as described for Figure 1B. (B) Caspase 3 activity was measured in primary mouse hepatocytes stimulated with either only TGF-β (5 ng/ml), EtOH (50, 100, 150 mM) or both for 48 h. The average activity of 3 independent experiments ± SEM is

shown. Values were normalized to the total protein content of each sample. RFU = relative fluorescence units. (C) Mouse hepatocytes treated with 5 ng/ml TGF-β1, 100 mM ethanol or a combination of both were stained with Annexin-V-Cy3 (red / apoptotic cells) and Hoechst 33342 (blue / nuclei). (D) Immunoblot demonstrating overexpression of BCL2. Primary cells were infected with either AdLacZ or AdBCL2, or kept uninfected (ctrl). Strong expression of BCL2 was detected in the AdBcl2 sample. (E) HCC tissue extracts (N=3) were analyzed for phospho-Smad2/3 levels upon TGF-β, ethanol or TGF-β+ethanol treatment via ELISA and calculated as increase relative to that of untreated control (ctrl). (F) Immunohistochemical detection of phospho-Smad2/3 in representative examples of untreated HCC tissuesNuclear accumulation of activated Smad2/3 could be demonstrated (400x).

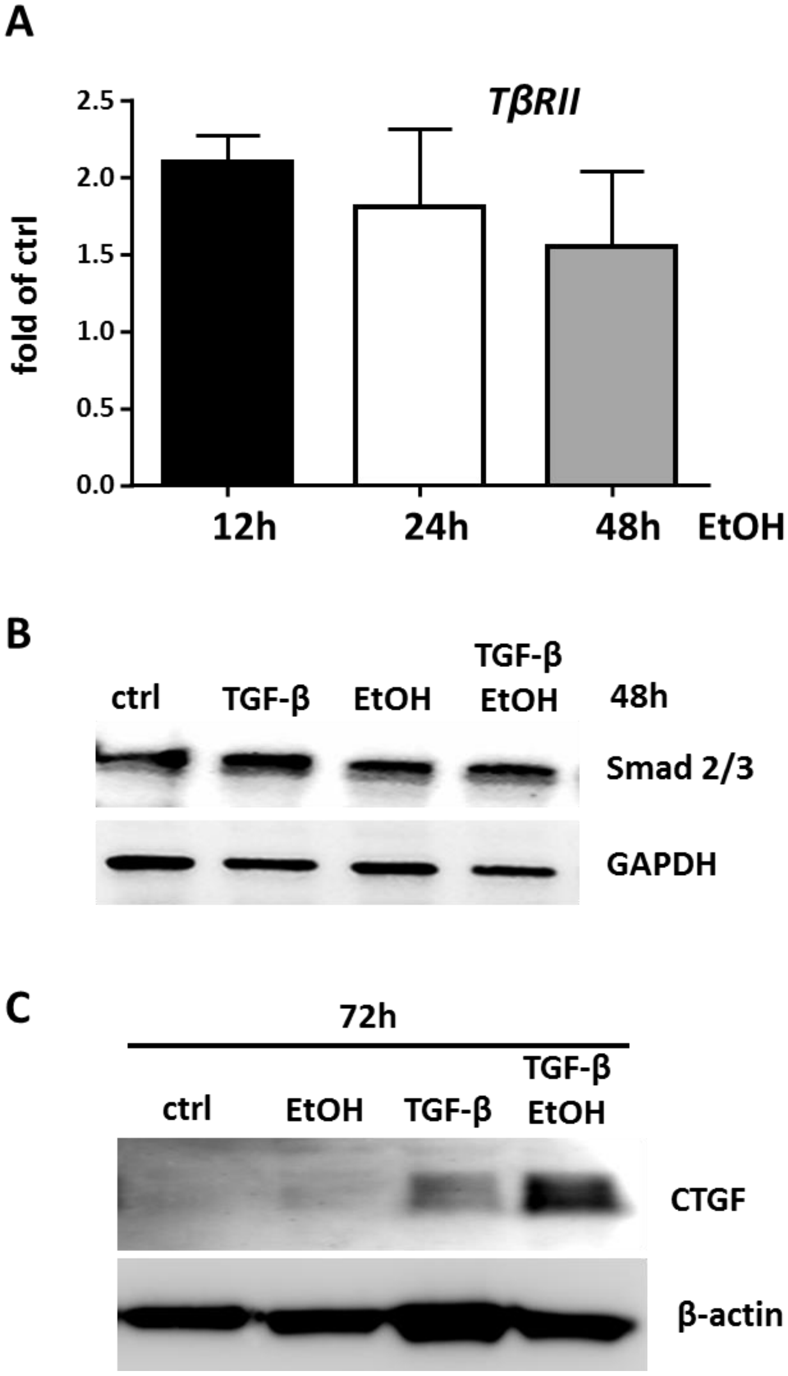


**Suppl. Figure 2**: (A) Mouse hepatocytes were incubated with ethanol (150 mM) for 12, 24, and 48 h. Subsequent qRT-PCR mRNA analysis revealed an increase in *TβRII* expression compared to untreated. (B) TGF-β/ethanol treatment (5ng/ml; 150 mM) effects on total Smad 2/3. (C) Immunoblot analysis of CTGF expression in mouse hepatocytes treated with TGF-β and ethanol as indicated and described in Figure 1B.

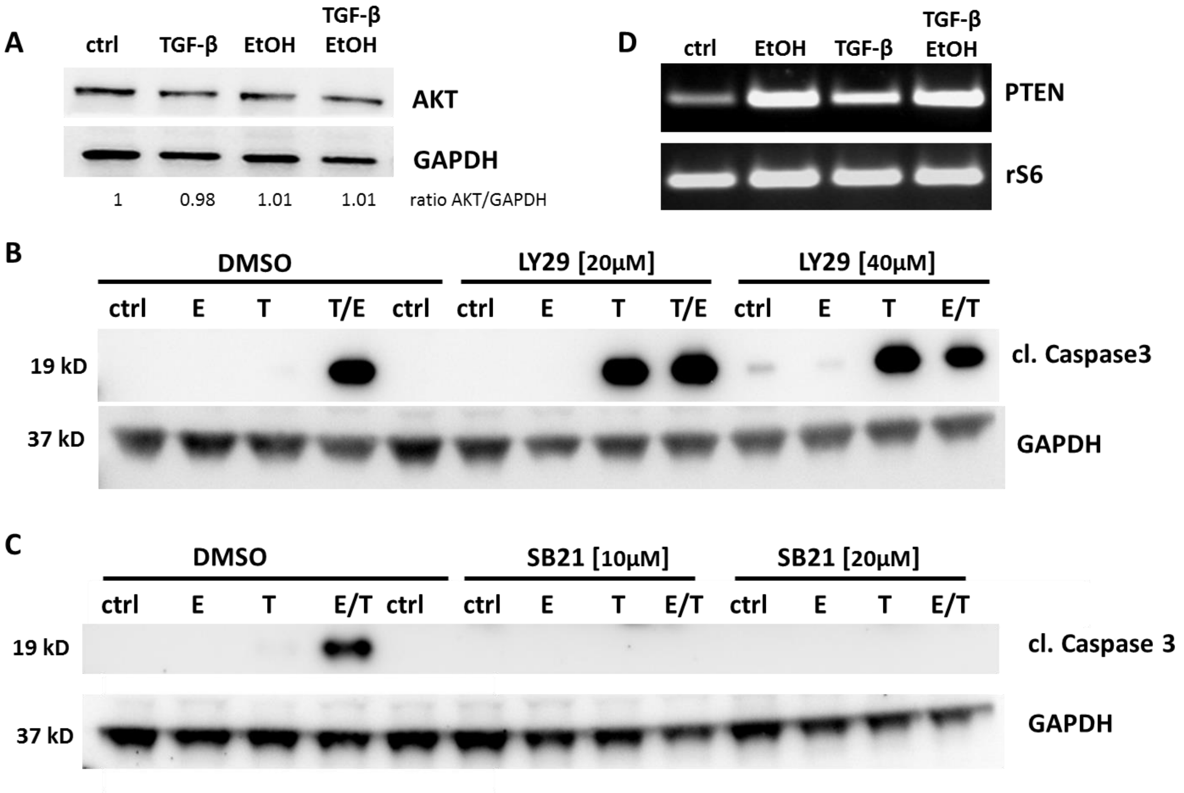


**Suppl. Figure 3**: (A) Total AKT expression upon 48 h treatment with TGF-β and/or ethanol. (B, C) Detection of cleaved caspase-3 by immunoblot analysis using hepatocyte lysates after 48 h treatment with 5ng/ml TGF-β (T) and/or 150 mM ethanol (E) and two different concentrations of the small molecule (B) AKT inhibitor LY294002 or (C) GSK3β inhibitor SB216763, as indicated. Detection of GAPDH was performed as loading control. (D) Conventional PCR analysis of *Pten* expression in mouse hepatocytes treated with TGF-β and ethanol as indicated, and described in Figure 1B. rs6 mRNA expression was used as loading reference.

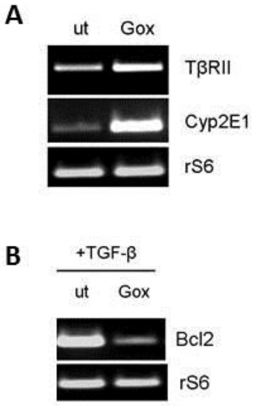


**Suppl. Figure 4**: Conventional PCR analysis of (A) *TβRII*, *Cyp2E1* and (B) *Bcl2* in mouse hepatocytes treated with TGF-β and GOX/CAT as indicated, and described in Figure 1B. rS6 mRNA expression was used as loading reference.

## Methods

### Detection of pSmad2/3 by ELISA

After incubation with TGF-β,ethanol or both, HCC liver tissues were homogenized in lysis buffer (Cell Signaling Technology, Danvers, MA, USA) and protein content was determined by bicinchoninic acid (BCA) protein assay kit (Thermo Scientific, Braunschweig, Germany). Phosphorylated Smad2/3 was detected in the liver tissue extracts by using thePATHSCAN ELISA kit (Cell Signaling Technology, Danvers, MA, USA) according to the manufacturer's instructions. Lysates were diluted 1:1 with sample diluent and incubated overnight at 4 °C in the appropriate well. After washing, the samples were incubated with a HRP-conjugated secondary antibody for 30 min at 37 °C. Following repeated washing, the TMB substrate was added for 30 min at room temperature. Finally, the reaction was stopped and absorbance of the samples was measured at 450 nm using a Tecan microplate reader (Tecan, Durham, USA). All samples were measured in duplicates.

### Detection of pSmad2/3 by immunohistochemistry

Paraffin sections of HCC tissues were deparaffinized in xylene and rehydrated through degrading concentrations of ethanol. Endogenous peroxidase was blocked with 3 % hydrogen peroxide in methanol. For antigen retrieval, slides were boiled for 5 minutes in antigen-unmasking solution (Vector Laboratories, Burlingame, CA, USA). After washing in 0.05 M Tris-HCl buffer (pH 7.4), nonspecific binding sides were blocked with blocking solution (VECTASTAIN ABC kit; Vector Laboratories) for 1 hour at room temperature. Afterwards, sections were incubated overnight at 4 °C with primary antibody against p-SMAD2/3 (1:100, Cell Signaling, #8828). After repeated washings with Tris buffer, sections were incubated with biotinylated secondary antibody solution (VECTASTAIN ABC kit; Vector Laboratories) for 30 minutes and covered with avidin-biotin complex reagent (VECTASTAIN ABC Kit; Vector Laboratories) for 1 hour at room temperature. Sections were stained in aminoethylcarbazole substrate solution (AEC chromogen kit; Sigma-Aldrich, St. Louis, MO, USA) and counterstained with hematoxylin. Finally, sections were covered with mounting medium (Aquamount, VWR International GmbH, Darmstadt, Germany) and micrographs were taken.

### Densitometry of AKT/GAPDH immunoblot

ImageJ software 1.49 was used to analyze immunoblot pictures of AKT and GAPDH. Positive bands were selected and processed via gel analysis. Subsequent quantification was processed in Excel.
